# Genome Sequences of Two Novel Coronavirus (SARS-CoV-2) Isolates from Dhaka, Bangladesh

**DOI:** 10.1128/MRA.00511-21

**Published:** 2021-07-08

**Authors:** M. Abdul Matin, Abdullah Al Nahid, Bishajit Sarkar, M. Asad Ullah, Yusha Araf, Nihad Adnan, Rashedul Islam, Mohammad Shahedur Rahman

**Affiliations:** a Department of Biotechnology and Genetic Engineering, Faculty of Biological Sciences, Jahangirnagar University, Savar, Dhaka, Bangladesh; b COVID Research Cell (CRC), Wazed Miah Science Research Center (WMSRC), Jahangirnagar University, Savar, Dhaka, Bangladesh; c Department of Biochemistry and Molecular Biology, School of Life Sciences, Shahjalal University of Science and Technology, Sylhet, Bangladesh; d Department of Genetic Engineering and Biotechnology, School of Life Sciences, Shahjalal University of Science and Technology, Sylhet, Bangladesh; e Department of Microbiology, Faculty of Biological Sciences, Jahangirnagar University, Savar, Dhaka, Bangladesh; f X-Genomics Research Laboratory, Dhaka, Bangladesh; KU Leuven

## Abstract

This study reports the genome sequences of two severe acute respiratory syndrome coronavirus 2 (SARS-CoV-2) strains detected in the nasopharyngeal swab specimens of two coronavirus disease 2019 (COVID-19) patients from Dhaka, Bangladesh.

## ANNOUNCEMENT

The ongoing coronavirus disease 2019 (COVID-19) pandemic is caused by the viral pathogen severe acute respiratory syndrome coronavirus 2 (SARS-CoV-2), a member of the *Betacoronavirus* in the *Coronaviridae* family (1). The virus was first detected in late December 2019 in Wuhan, Hubei Province, China (2). On 8 March 2020, the first three cases of COVID-19 in Bangladesh were identified. As of 18 May 2021, a total of 780,857 confirmed cases of the disease with 12,181 deaths had been reported in the country (3). We announce here two genome sequences of SARS-CoV-2 from the collected samples of two female patients, 43 and 28 years old. The samples were collected on 2 and 4 December 2020 during the time period of a relatively reduced number of confirmed COVID-19 cases in Bangladesh (https://www.worldometers.info/coronavirus), prior to the second wave of the pandemic in the country from late March to early April 2021 (4). Both patients provided informed consent and were found to be COVID-19 positive by reverse transcription-PCR (RT-PCR) (5). The Ethical Review Board of Jahangirnagar University [reference no. BBEC, JU/M 2020/COVID-19/(8)3] approved all the protocols used to carry out this study.

The viral nucleic acids were extracted from the collected nasopharyngeal swab specimens using the ReliaPrep viral TNA miniprep system (catalog no. AX4820; Promega) according to the manufacturer’s protocol. Library preparation was performed using an Illumina RNA prep kit with RNA enrichment Respiratory Virus Oligo Panel following the manufacturer’s instructions. Thereafter, an Illumina MiniSeq system was used according to the manufacturer’s protocol to sequence the prepared libraries in the paired-end format with a read length of 74 bp.

**FIG 1:**
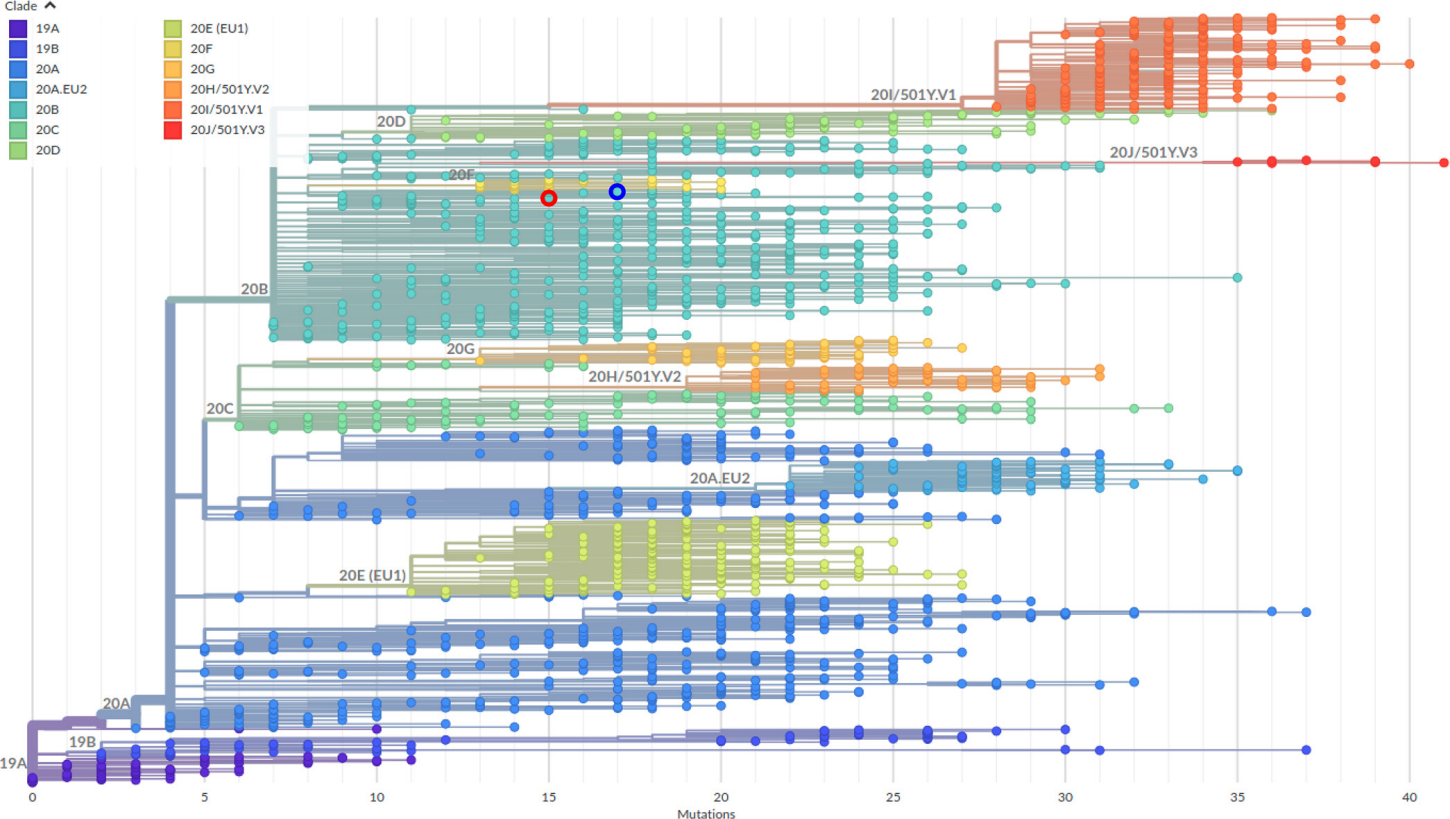
The genomes isolated from the two SARS-CoV-2 samples from the Dhaka District of Bangladesh are presented on a phylogenetic tree. The number of mutations in comparison to the reference genome of the Wuhan-Hu-1 isolate (GenBank accession no. MN908947.3) is represented by the *x* axis. Nextclade beta version 0.14.2 (clades.nextstrain.org) was used to construct the tree on 4 May 2021, with the red and dark blue circles representing the positions of the hCoV-19/Bangladesh/JU-WMSRC-Dhaka-5/2020 (GISAID accession no. EPI_ISL_884088) and hCoV-19/Bangladesh/JU-WMSRC-Dhaka-8/2020 (GISAID accession no. EPI_ISL_884087) sequences, respectively.

A total of 7,170,754 and 150,934 reads, respectively, from the SARS-CoV-2 samples were generated and quality assessed with FastQC version 0.11.8 (6). The raw reads were adapter trimmed with Trimmomatic version 0.39 (7), and the surviving 6,716,784 and 142,054 reads were subsequently mapped to the SARS-CoV-2 reference genome (GenBank accession no. MN908947.3) using the Burrows-Wheeler Aligner (BWA) version 0.7.17 (8). Ultimately, the consensus genomes were generated from the mapped reads using SAMtools version 1.11 (9). The genomes of the two Bangladeshi SARS-CoV-2 samples, i.e., hCoV-19/Bangladesh/JU-WMSRC-Dhaka-5/2020 and hCoV-19/Bangladesh/JU-WMSRC-Dhaka-8/2020, were found to have base pair lengths of 29,888 and 29,848, respectively, with an average coverage of over 4,313× and 215×, respectively. No indels were found, and the GC content was measured to be 38% for both genomes. The consensus genomes along with the related sample metadata were uploaded to the Global Initiative on Sharing All Influenza Data (GISAID) database (10) on 27 January 2021. Nextclade beta version 0.14.2 (clades.nextstrain.org) by Nextstrain (11) was utilized in the phylogenetic analysis, which assigned both sequences to SARS-CoV-2 clade 20B (Fig. 1). The closest ancestral strains of the genomes include sequences from Bangladesh (GISAID accession no. EPI_ISL_959367, EPI_ISL_943554, and EPI_ISL_959386) and New Zealand (GISAID accession no. EPI_ISL_682280) according to Nextclade.

Genome variation analysis of the consensus genomes was performed using the Genome Detective Virus Tool version 1.132 (12), which indicated several changes in the studied genomes relative to the SARS-CoV-2 reference genome sequence of the Wuhan-Hu-1 isolate (GenBank accession no. NC_045512.2). The sequence of the hCoV-19/Bangladesh/JU-WMSRC-Dhaka-5/2020 strain exhibited 6 synonymous and 9 nonsynonymous mutations, whereas 7 synonymous and 10 nonsynonymous mutations were observed in the sequence of the hCoV-19/Bangladesh/JU-WMSRC-Dhaka-8/2020 strain (Table 1). Except for the Q94L mutation in the ORF7a gene, all nonsynonymous mutations were in both strains. According to the Nextstrain community build of Bangladesh (https://nextstrain.org/community/CHRF-Genomics/ncovBangladesh@main), the 9 nonsynonymous mutations common to the two studied genomes are also prevalent in other Bangladeshi SARS-CoV-2 genome sequences. However, the ORF7a Q94L mutation could not be detected in any other Bangladeshi genomes as of 4 May 2021. The most common mutations observed in SARS-CoV-2 genomes from Bangladesh include 5 nonsynonymous mutations that were also found in both studied genomes—I300F and P4715L in the ORF1ab gene, D614G in the S gene, and R203K and G204R in the N gene (13).

**TABLE 1:** Mutations detected in both the hCoV-19/Bangladesh/JU-WMSRC-Dhaka-5/2020 (strain A) and hCoV-19/Bangladesh/JU-WMSRC-Dhaka-8/2020 (strain B) sequences compared to the reference genome of the Wuhan-Hu-1 isolate (GenBank accession no. MN908947.3)

Strain(s)	Nucleotide position	Reference nucleotide	Mutated nucleotide	Gene	Amino acid change
A, B	241	C	T	5′-UTR	Noncoding
A, B	335	C	T	ORF1ab	R24C
A, B	434	G	A	ORF1ab	E57K
A, B	1163	A	T	ORF1ab	I300F
B	1333	T	G	ORF1ab	None (synonymous mutation)
A, B	3037	C	T	ORF1ab	None (synonymous mutation)
A, B	3652	A	G	ORF1ab	None (synonymous mutation)
A	6229	A	G	ORF1ab	None (synonymous mutation)
A, B	9436	A	G	ORF1ab	None (synonymous mutation)
A, B	14408	C	T	ORF1ab	P4715L
B	18744	C	T	ORF1ab	None (synonymous mutation)
A	21907	G	A	S	None (synonymous mutation)
B	22801	G	T	S	None (synonymous mutation)
A, B	23403	A	G	S	D614G
A, B	23604	C	G	S	P681R
B	27674	A	T	ORF7a	Q94L
A, B	28881	G	A	N	R203K
A, B	28882	G	A	N	R203K
A, B	28883	G	C	N	G204R

As more genomes of COVID-19 patients with various clinical features from various regions of the country are being sequenced on a regular basis, the evolution of the virus must be further investigated and closely monitored in order to track COVID-19 progression in Bangladesh.

### Data availability.

The SARS-CoV-2 genome sequences from Bangladesh were deposited in the GISAID database (accession no. EPI_ISL_884087 and EPI_ISL_884088), and the raw reads have been submitted to the NCBI Sequence Read Archive (SRA). The BioProject accession no. is PRJNA701872 for both samples. The BioSample and SRA accession no. for the samples are SAMN17915311 and SAMN17915310 (BioSample) and SRR13722033 and SRR13721921 (SRA).
